# Testosterone- and Cortisol-Secreting Adrenocortical Oncocytoma: An Unusual Cause of Hirsutism

**DOI:** 10.1155/2014/206890

**Published:** 2014-03-11

**Authors:** Serap Baydur Sahin, Ahmet Fikret Yucel, Recep Bedir, Sabri Ogullar, Teslime Ayaz, Ekrem Algun

**Affiliations:** ^1^Department of Endocrinology and Metabolism Disease, Recep Tayyip Erdogan University Medical School, 53020 Rize, Turkey; ^2^Department of Surgery, Recep Tayyip Erdogan University, 53020 Rize, Turkey; ^3^Department of Pathology, Recep Tayyip Erdogan University, 53020 Rize, Turkey; ^4^Department of Radiology, Recep Tayyip Erdogan University, 53020 Rize, Turkey; ^5^Department of Internal Medicine, Recep Tayyip Erdogan University Medical School, 53020 Rize, Turkey

## Abstract

*Objective.* Oncocytomas of the adrenal cortex are usually benign and nonfunctional. They are rarely seen as the cause of hirsutism. Therefore, we aimed to report a case of adrenocortical oncocytoma presenting with hirsutism. *Methods.* We report a testosterone- and cortisol-secreting adrenal oncocytoma in a 23-year-old female patient presenting with hirsutism. *Results.* The patient had the complaint of hirsutism for the last year. Laboratory tests revealed total testosterone level of 4.2 ng/mL, free testosterone of >100 pg/mL, and DHEAS level of 574 µg/dL. There was no suppression in cortisol levels with 2 mg dexamethasone suppression test (5.4 µg/dL). Adrenal MRI revealed a 27 × 25 mm isointense solid mass lesion in the left adrenal gland and the patient underwent laparoscopic left adrenalectomy. Pathological examination confirmed the diagnosis of benign adrenocortical oncoyctoma. *Conclusion.* This well-characterized case describes a testosterone- and cortisol-secreting adrenocortical oncocytoma as a possible cause of hirsutism. To our knowledge, this is the second report in the literature. Adrenal oncocytomas should always be considered in the differential diagnosis of hirsutism.

## 1. Introduction

Hirsutism, defined as excessive male-pattern hair growth, affects between 5 and 10% of women of reproductive age and most women with hirsutism have polycystic ovary syndrome [1, 2]. Androgen-secreting tumors are rarely seen as the cause of hirsutism. In an epidemiological study, the frequency of androgen-secreting tumors was 0.2% in 950 hirsute women [3]. Most testosterone-secreting tumors arise from the ovary and rarely origins from the adrenal gland.

Oncocytic neoplasms or oncocytomas usually arise in the kidneys or thyroid, parathyroid, salivary, or pituitary glands [4]. Oncocytomas of the adrenal cortex are extremely rare and usually detected incidentally [5]. Adrenal oncocytomas are usually benign and nonfunctional in most of cases. Herein, we report a testosterone- and cortisol-secreting adrenal oncocytoma in a 23-year-old female patient presenting with hirsutism.

## 2. Case Report

A 23-year-old female patient admitted to endocrinology outpatient clinic with the complaint of hirsutism for the last year. Excessive hair growth was identified to originate from facial and mandibular areas initially and then to spread to abdominal and thoracic regions. She had regular menstrual cycles since her first period by the age of 12. Medical background and family history were unremarkable. Physical examination revealed that body temperature was 37°C, pulse rate was 80 beats/min, height was 156 cm, weight was 61 kg, BMI was 25.4 kg/m^2^, and the blood pressure was 120/80 mm Hg. She had acnes on her face while showed no signs of moon face or facial plethora. Thyroid gland was nonpalpable and abdominal examination was normal in superficial and deep palpation. No organomegaly or mass was detected. Examination of the urogenital system revealed normal findings with feminine type of hair growth and normal breast development. Absence of ecchymosis, normal turgor and tonus, and normal skin thickness were noted in the examination of the skin. There were no findings of purple stria and acanthosis nigricans, whereas hirsutism was remarkable. Ferriman Gallwey score was 23. There were no signs of virilization including vocal changes, muscular hypertrophy, breast atrophy, and hypertrophy of clitoris.

**Figure 1:**
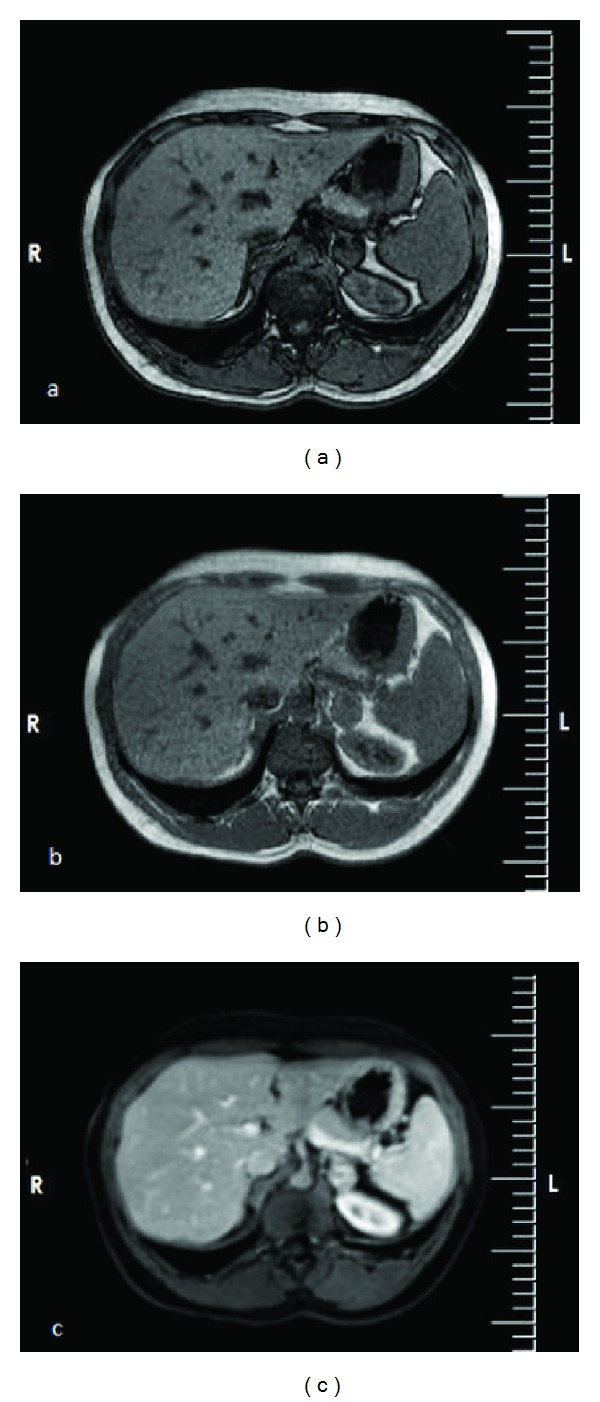
(a–c) Axial T1-weighted out-phase MR images (a), in-phase MR image (b), and three-dimensional GRE contrast-enhanced MR image show the intense mass in left adrenal gland. (c) shows no loss of signal intensity on the out-of-phase image.

Given the findings of marked hirsutism and facial acne, laboratory tests were performed for the differential diagnosis of hirsutism which revealed total testosterone level of 4.2 ng/mL, free testosterone of >100 pg/mL, and DHEAS level of 574 *μ*g/dL (Table 1). Pelvic USG revealed normal endometrial thickness besides normal size of uterus and normal size and appearance of ovaries. Based on these findings, the likelihood of an androgen-secreting adrenal tumor was considered in the initial diagnosis and, therefore, the adrenal MRI was performed. MRI revealed a 27 × 25 mm isointense solid mass lesion in the left adrenal gland in T1A and T2A series (Figure 1). Afterwards, functional screening for adenoma was performed which revealed normal findings on a 24-hour urine test for metanephrine (73.91 mcg/day) and normetanephrine (133.95 mcg/day) besides normal levels for aldosterone (65 pg/mL) and renin (2.1 ng/mL). Lacking findings related to Cushing syndrome in the physical examination, our patient had basal ACTH levels of <5 pg/mL twice. Dexamethasone (1 mg) suppression test (DST) was 4.7 *μ*g/dL and there was no suppression in cortisol levels also with 2 mg DST (5.4 *μ*g/dL). Urinary cortisol level was normal (75 mcg/day), while the physiological cortisol circadian rhythm was determined to be disturbed.

**Figure 2:**
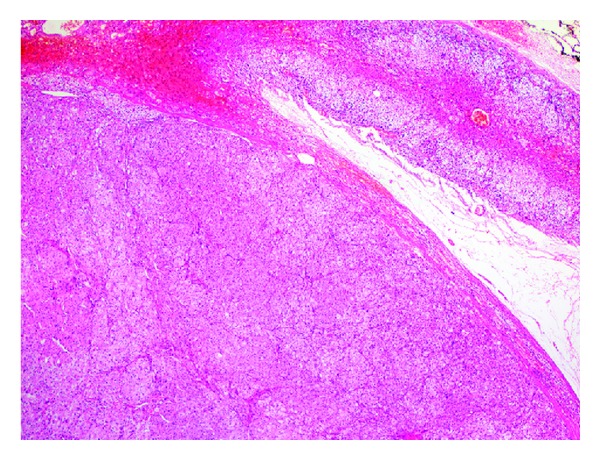
Compressed adrenal cortical tissue is noted at the periphery. The tumor is a solid growth of neoplastic cells with eosinophilic cytoplasm (H&E × 40).

**Figure 3:**
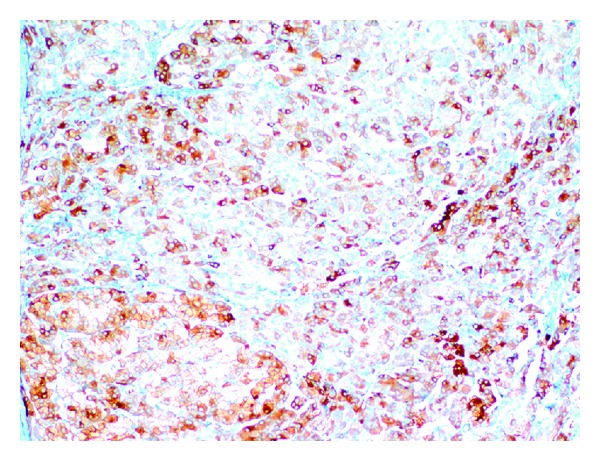
Immunohistochemical study showing diffuse positive staining for synaptophysin (immunohistochemistry, x100).

Based on consideration of overall findings, with the initial diagnosis of testosterone- and cortisol-secreting tumor in the left adrenal gland, the patient underwent laparoscopic left adrenalectomy with perioperative steroid replacement. Pathological examination confirmed the diagnosis of benign adrenocortical oncocytoma. Macroscopically, the tumor was a rounded, encapsulated, and well-circumscribed mass, with an average diameter of 2.2 × 2 cm. The microscopic appearance of oncocytic neoplasm included cells with eosinophilic cytoplasm arranged in solid pattern (Figure 2). Immunohistochemical studies revealed that the case was positive for CD56, synaptophysin (Figure 3), and vimentin and negative for chromogranin. Ki-67 proliferation index was found 3% in the tumor. Capsular and venous invasion, high mitotic rate, and atypical mitoses were not observed, so it was considered as benign according to the Lin-Weiss-Bisceglia criteria [6]. At the postoperative 72nd hour, cortisol level was 15.7 mcg/dL at 08:00 a.m., while ACTH level was 7.66 pg/mL. There was no need for postoperative steroid treatment. Postoperative total testosterone level was determined to regress to 1.05 ng/mL, free testosterone to 2.26 pg/mL, and DHEAS to 345.6 mcg/dL.

## 3. Discussion

Oncocytic neoplasms arising in the adrenal glands are extremely rare. Although exact overall incidence is unknown, approximately 147 cases have been reported in the literature [5]. They have been reported in patients across a wide range of ages (3–77 years) with a significant female to male predominance [7]. The neoplasms varied in size from 2.2 cm to 15 cm [8].

The adrenal oncocytomas are usually nonfunctional. They are usually discovered incidentally, although rarely present associated with hormone-related symptoms. In cases with androgen secretion, the disease has sudden onset and characteristic clinical features including hirsutism, acne, frontal hair loss, and ovulatory disorders. Our patient also presented with hirsutism showing high serum testosterone and DHEAS levels. Very rare cases of androgen-secreting adrenocortical oncocytic neoplasms have been reported [9–13]. The other clinical presentation may be Cushing's syndrome and, in the literature, 4 cases have been reported [8, 14–16].

Our patient had a testosterone-producing adrenocortical oncocytoma and also was demonstrated to secrete cortisol. She did not have the clinical signs of Cushing's syndrome; however, the laboratory findings supported the diagnosis of syndrome. It may be related to milder elevated glucocorticoid secretions. As a result, we thought that our patient had a functional adrenocortical oncocytoma leading to hirsutism and subclinical Cushing's syndrome. Although clinically silent, in 5–20% of cases, adrenal incidentalomas are responsible for a subtle cortisol overproduction, commonly defined as “subclinical Cushing's syndrome.” It is assumed that glucocorticoid production in these patients is insufficient to cause a clinically recognizable syndrome [17]. To our knowledge, testosterone- and cortisol-producing adrenocortical oncocytoma has been reported so far in only 1 case [18]. This case who was a 58-year-old postmenopausal woman had poor glycemic and blood pressure control. She had male pattern hair loss and high testosterone and DHEA-S levels. She failed to suppress plasma cortisol after 1 mg DST. A computed tomography scan revealed an 87 × 88 mm mass at the right adrenal gland. Histology confirmed the diagnosis of benign adrenocortical oncocytoma similarly with our findings. The tumour had no evidence of necrosis, invasion, or metastases. Immunohistochemistry showed the tumour cells to be positive for synaptophysin, vimentin, and melan-A and negative for chromogranin. These results were similar to our patient's histopathological findings.

The adrenocortical oncocytomas are mostly benign tumors. They have their own structural features. The microscopic appearance of oncocytic cells is highly eosinophilic and granular and the immunophenotypic profile is diffuse positivity for vimentin, melan-A, synaptophysin, and alpha-inhibin in general [5]. In our patient, the neoplasm was diffuse positive for vimentin and synaptophysin. To distinguish benign adrenocortical neoplasms from the malignant ones, the Lin-Weiss-Bisceglia criteria are used [6]. According to these criteria, high mitotic rate, atypical mitoses, and venous invasion are defined as major criteria and large size and huge weight, necrosis, capsular invasion, and sinusoidal invasion are defined as the minor criteria. All major and minor criteria were absent in our patient; therefore, our patient was diagnosed as benign oncocytic neoplasm according to the system proposed by Bisceglia et al.

## 4. Conclusion

Although it is rare, testosterone-secreting adrenal oncocytomas should always be considered in the differential diagnosis of hirsutism and also all the hormonal analyses must be undertaken to rule out the presence of other hormone secretions caused by the adrenal mass.

## Figures and Tables

**Table 1:** Hormone measurements in the patient.

Parameter	Result	Reference range
ACTH	<5	0–46 pg/mL
Cortisol	12.3	3.7–19.4 µg/dL
FSH	5.8	3.03–8.08 mIU/mL
LH	4.89	1.8–11.78 mIU/mL
Estradiol	21	18–147 pg/mL
SHBG	41.6	26.10–110 nmol/L
Prolactin	10.2	5.18–26.53 ng/mL
Total testosterone	4.2	0.09–1.3 ng/mL
Free testosterone	>100	1.1–3.1 pg/mL
DHEAS	574	35–430 µg/dL
17-OH progesterone	1.9	0.1–1 ng/mL
FT3	3.4	1.71–3.71 pg/mL
FT4	1.2	0.7–1.48 ng/dL
TSH	0.6	0.35–4.94 uIU/mL
